# Quality Improvement to Promote Advance Care Planning for Young Adults with Duchenne Muscular Dystrophy

**DOI:** 10.1177/26892820261422094

**Published:** 2026-04-21

**Authors:** Clara Mao, Lori Herbst

**Affiliations:** 1 Department of Internal Medicine, University of Cincinnati College of Medicine, Cincinnati, Ohio, USA.; 2 Department of Pediatrics, Cincinnati Children’s Hospital Medical Center, Cincinnati, Ohio, USA.; 3 Department of Anesthesia, Division of Pain Management and Palliative Medicine, Cincinnati Children’s Hospital Medical Center, University of Cincinnati College of Medicine, Cincinnati, Ohio, USA.; 4 Department of Pediatrics, Division of Hospital Medicine, University of Cincinnati College of Medicine, Cincinnati Children’s Hospital Medical Center, Cincinnati, Ohio, USA.

**Keywords:** advance directives, advance care planning, code status, Duchenne muscular dystrophy, end-of-life care, quality improvement

## Abstract

**Background:**

Duchenne muscular dystrophy (DMD) is a progressive neuromuscular disease. With improved life expectancy of patients with DMD, the need for advance care planning (ACP) has been identified by patients and providers. A quality improvement effort to address this gap was initiated at an academic children’s hospital where patients are seen within a multidisciplinary neuromuscular clinic, which integrates palliative care into visits starting at age 18. The team followed outcome measures of (1) health care power of attorney (HCPOA) paperwork completion and (2) code status order documentation from baseline rates of 29% and 6%, respectively, between August 2024 and August 2025 for patients with DMD aged 18 or older.

**Methods:**

Identified key drivers included provider awareness of prior ACP conversations, documentation workflow, and patient readiness for conversations. Interventions included: creation of a DMD ACP Checklist, provider education, weekly e-mails, and access to state-specific ACP documents.

**Results:**

123 visits were reviewed over 13 months. Improvement interventions led to a centerline shift for both outcome measures, with an increase from 29% to 68% for HCPOA paperwork completion and from 6% to 72% for code status order documentation.

**Conclusions:**

This project demonstrated that many young adults with DMD were open to ACP conversations, as reflected in improved rates of HCPOA paperwork completion and code status order documentation. However, some families continued to express discomfort with topics surrounding end-of-life. Further exploration is needed to understand how to best address these challenges in order to provide goal-concordant care with disease progression.

## Introduction

Duchenne muscular dystrophy (DMD) is a progressive, life-limiting neuromuscular disorder. During childhood, patients become nonambulatory and are faced with complications including respiratory weakness and cardiomyopathy.^1,2^ Many require respiratory support as pulmonary function declines, including nocturnal and daytime noninvasive positive pressure ventilation (NIPPV).^1,2^ Recent advancements in care have resulted in a longer life expectancy for patients with DMD, with individuals now living into their third and fourth decades. As patients survive further into adulthood, a need for improved transitions of care and advance care planning (ACP) has been identified by patients, families, and providers.^3^ ACP involves the process of planning for future medical decisions by discussing goals and values guiding care and by completing advance directives. Despite the importance of ACP for young adults with DMD, data suggests that these conversations are not being routinely held, and there remains limited research to guide clinicians on how and when to initiate them.^4^

High symptom burden and presence of comorbidities affect the quality of life of patients with DMD.^5^ Palliative care providers can assist in symptom management and ACP, but are frequently underutilized in DMD care.^6^ A qualitative study of interviews conducted with patients suggested that conversations are deferred due to the gradual progression of disease and a focus on living in the present.^6^ Some patients with DMD struggle with making medical decisions as they enter adulthood because they are used to relying on caregivers to direct care.^7^ Others view ACP as an opportunity to establish autonomy by clearly documenting treatment preferences.^8^ Many are open to exploring concepts of end-of-life care but defer to their clinicians to be proactive about bringing up those topics.^4,6,9^ One successful example at a clinic caring for young adults with neuromuscular disorders utilized a conversation tool to initiate ACP discussions and increase advance directive documentation.^10^

Over recent years, the palliative care team at a large academic children’s hospital was integrated into the multidisciplinary neuromuscular clinic, where patients with DMD continue to be seen into adulthood. By framing palliative care involvement as routine, the aim was to promote conversations in a familiar outpatient environment during a more stable period of disease. However, the team identified that despite consistent palliative care presence in clinic, there was a lack of standardized approach to ACP discussions. Patients continued to lack completed health care power of attorney (HCPOA) paperwork and clearly documented care preferences including code status, thus quality improvement (QI) efforts were implemented to improve these outcomes.

## Methods

### Context

Patients with DMD seen in the outpatient neuromuscular clinic of an urban, free-standing, academic children’s hospital in the midwestern United States receive standard palliative care consults starting at age 18. Clinics are held weekly. During clinic visits, patients see subspecialists from neurology, cardiology, pulmonology, palliative care, physical medicine and rehabilitation, psychology, nutrition, social work, physical therapy, and occupational therapy. The patient population includes patients from the local catchment area and patients who travel from other states. A palliative care physician or advance practice provider joins the clinic each week and sees anywhere from one to five patients.

### Improvement team

**FIG. 1.**
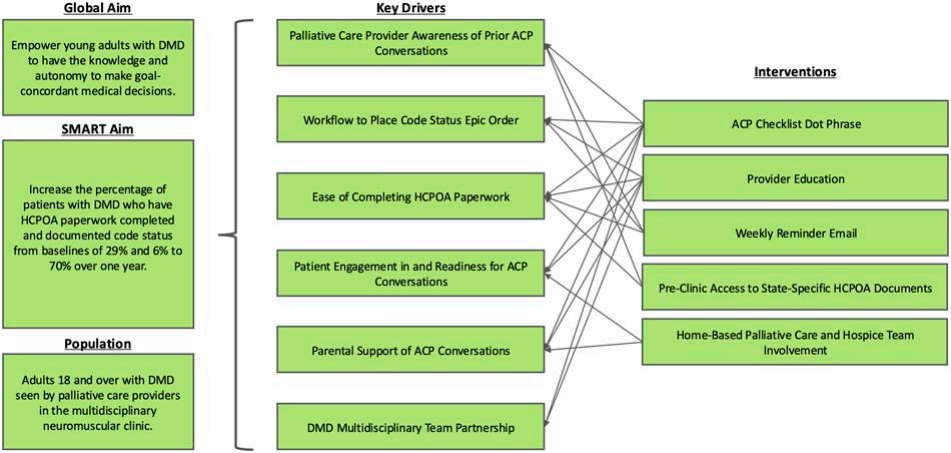
Key driver diagram of concepts targeted to achieve aims of the quality improvement effort. ACP, advance care planning; DMD, Duchenne muscular dystrophy; HCPOA, health care power of attorney; SMART, specific, measurable, achievable, relevant, time-based.

The improvement team included palliative care attendings and advanced practice providers, as well as an internal medicine–pediatrics resident. Additional input was sought from key stakeholders including parents of young adults with DMD, subspecialty physicians, the clinic flow lead, and social workers. The team created a process map and performed a systems failure mode and effects analysis to identify key drivers (Fig. 1).

### Interventions

Interventions were developed and implemented through Plan-Do-Study-Act cycles. They included: (1) creation of a DMD ACP Checklist for documentation, (2) provider education, (3) weekly reminder e-mails, (4) access to state-specific documents, and (5) involvement of the home-based palliative care and hospice team.

#### DMD ACP checklist

To better standardize ACP topics addressed during palliative care visits, a checklist was developed using an iterative process from end-user feedback to be included within clinic notes using a dot phrase (Table 1). Prior to the intervention, team members noted spending the majority of visits providing general education on ACP topics as opposed to further exploring specific preferences related to disease progression. The checklist was designed to improve clarity on prior conversations and document progress on goal development to allow for continuity across care settings and between providers. In the checklist, providers were able to indicate the date each topic was discussed and the current status of conversations. Topics included introduction of palliative care, HCPOA, code status, respiratory failure management, tracheostomy and mechanical ventilation, heart failure management, and ACP progress. The checklist included a smart link to the code status order active in the electronic health record (EHR) as a reminder of whether or not a code status order had previously been placed. Within this hospital system, it is not a requirement to have a code status order placed in either the inpatient or outpatient setting. Providers were able to indicate if they shared conversation tools such as Five Wishes, Voicing My Choices, The Conversation Project, and The DMD Transition Toolkit.^11–14^

**Table 1.** Duchenne Muscular Dystrophy Advance Care Planning Checklist

Topic	Date completed
Initial Visit	
Introduction of Palliative Care	
Decision Making	
Health Care Power of Attorney Education Provided—select below:	
Yes	
No	
Health Care Power of Attorney Completed—select below:	
Completed in clinic	
Completed previously, scanned in chart	
Completed previously, not scanned in chart	
Not completed	
Uncertain, will check at home	
Surrogate Decision Maker—select below:	
Legal next of kin	
Health care power of attorney	
Guardian	
Unknown	
None	
Contingency Planning Discussion	
Code Status: order auto-populates from electronic medical record if placed	
Respiratory failure and management	
Tracheostomy and mechanical ventilation	
Heart failure and management	
Resources Provided—select below:	
Health care power of attorney paperwork	
Living will	
Five Wishes	
Voicing My Choices	
The Conversation Project	
DMD Transition Toolkit	
Other	
None	
Advance Care Planning Progress—select below:	
Patient engaged, conversations ongoing	
Patient engaged, HCPOA and goals of care identified	
Patient not ready to discuss, routine follow-up in future	
Patient not ready to discuss, will plan covisit with neuromuscular clinic team member	
Patient declines palliative care involvement, please reconsult if needs arise	

DMD, Duchenne muscular dystrophy; HCPOA, health care power of attorney.

#### Provider education

Palliative care providers were educated on the goals of the QI project throughout the year during quarterly staff meetings by L.H., beginning at the end of July 2024. The team started by identifying gaps in understanding regarding key components of the project with regard to HCPOA paperwork, code status orders, supportive decision making, the DMD Transition Toolkit, and common reasons for decline in patients with DMD.^14–15^ Education focused on topics including: differences between HCPOA and legal next of kin, specific logistics of completing HCPOA paperwork, value of documenting code status in the EHR to indicate patient wishes to their broader health care team, and the concept of framing ACP as a tool to promote autonomy and empowerment.

#### Weekly reminder e-mails

The palliative care provider assigned to neuromuscular clinic changes weekly, resulting in variability in familiarity with the QI project goals and individual patients. Weekly e-mail reminders were sent by C.M. or L.H. and included pertinent information about prior palliative care discussions, status of HCPOA paperwork, code status, and electronic documents of state-specific ACP forms.

#### Access to state-specific documents

As a quaternary referral center, this institution cares for patients who travel from across the United States. State-by-state variability in ACP forms presents a challenge to access. While forms are available online, some can be difficult to find, which can be a barrier during busy clinic days. The authors addressed this issue through preclinic chart review to identify the home state of patients and determine the appropriate HCPOA form to send in advance.

#### Home-based palliative care and hospice teams

Local patients with more advanced DMD are frequently enrolled in the institution’s home-based program. Although only data from neuromuscular clinic visits were included in analysis, home-based palliative care and hospice social workers were encouraged to continue ACP in the home setting, as this allowed additional touchpoints for patients with more advanced disease. L.H. held an educational session reviewing steps of completing Ohio HCPOA paperwork, as pediatric team members lacked comfort completing HCPOA paperwork due to the age of their typical patients. Because the social workers often have longitudinal relationships with enrolled patients, they were uniquely in a position to help guide families through decisions when hesitancy was identified in the clinic setting.

### Measures

Outcome measures included the percentage of adults with DMD seen by palliative care in neuromuscular clinic each month with (1) self-reported completion of HCPOA documents and (2) code status order placed in the EHR. Outcome data were extracted from the EHR on retrospective chart review by C.M. Data collection were verified by L.H. for the first month to ensure reliability of the chart review process. Baseline data were collected from January 2024 through July 2024 during the run-in phase when the QI project was being conceptualized but not yet actively implemented. Intervention data were tracked over 13 months from August 2024 through August 2025. A goal of 70% was set for both outcome measures as this reflected a significant improvement from the baseline completion rates and allowed for patients to opt out.

### Analysis

Run charts were used to track outcome measures each month and run chart rules were followed to determine shifts in the centerline.^16^

### Ethical considerations

This project was designed to improve local institutional practices and quality of care, and not to create generalized knowledge. As such, it does not qualify as human subjects research requiring institutional review board review.

## Results

**FIG. 2.**
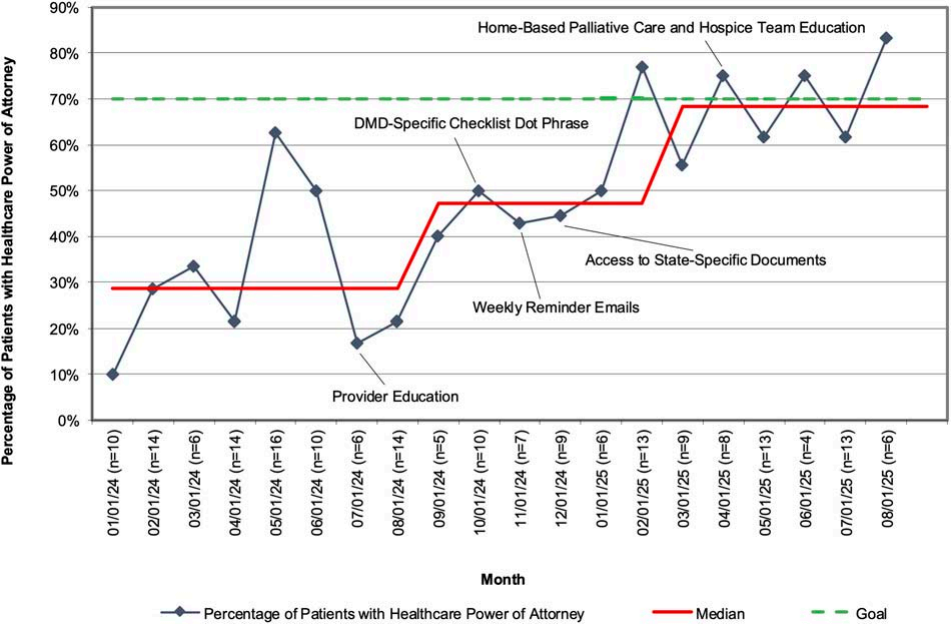
Run chart of percentage of patients with completed health care power of attorney paperwork.

**FIG. 3.**
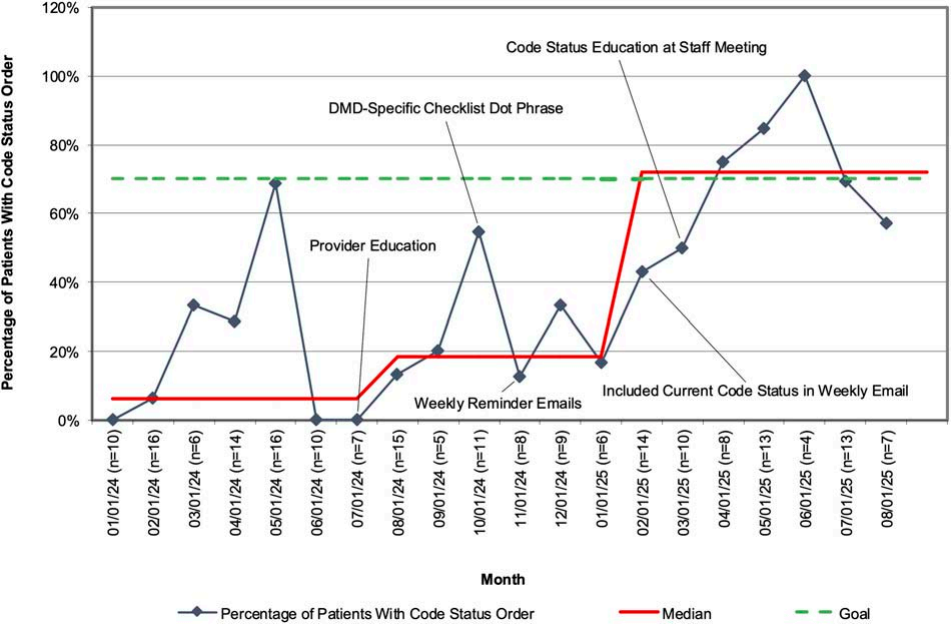
Run chart of percentage of patients with code status order placed in the electronic health record.

A total of 202 patient encounters were included, with 79 in the baseline period and 123 in the intervention phase. Age at patient encounters ranged from 18 to 34-years-old with a median of 23 (interquartile range 20–25). Additional data on demographics and clinical characteristics of the intervention group are summarized in Table 2. As expected at this age, most patients were nonambulatory and used NIPPV. In nine encounters, patients had legal guardians and were excluded from HCPOA completion data, as decision-making defaults to the legal guardian. Improvement interventions led to a centerline shift for both outcome measures, with an increase from 29% to 68% for completed HCPOA paperwork (Fig. 2) and from 6% to 72% for code status order placement (Fig. 3).

**Table 2.** Demographics and Clinical Characteristics of Young Adults with Duchenne Muscular Dystrophy

Characteristic	No. (%)
Race	
White	112 (91.1)
Native Hawaiian and Other Pacific Islander	3 (2.4)
Black or African American	2 (1.6)
Asian	2 (1.6)
Biracial	1 (0.8)
Not Available	3 (2.4)
Ethnicity	
Non-Hispanic	112 (91.1)
Hispanic	10 (8.1)
Not available	1 (0.8)
Language	
English	119 (96.7)
Spanish	3 (2.4)
Russian	1 (0.8)
Ambulation status	
Nonambulatory	111 (90.2)
Household ambulatory only	7 (5.7)
Household ambulatory and community ambulatory	5 (4.1)
Respiratory support	
Nocturnal noninvasive positive pressure ventilation only	85 (69.1)
Nocturnal and daytime noninvasive positive pressure ventilation	31 (25.2)
None	7 (5.7)

Of the patients who had a code status order placed in the intervention phase, 46% (27/59) were Full Code and 54% (32/59) had Do Not Resuscitate (DNR) orders. In the baseline phase, 18% (3/17) of code status orders were Full Code. Out-of-hospital DNR paperwork was provided to patients who selected a DNR order unless declined. Notably, only 3% (4/123) of patients were identified as not ready for or not interested in ACP conversations, with recommended follow-up plan clearly documented. Forty-nine percent of patients with completed HCPOA paperwork during the intervention phase had documents uploaded into the EHR (32/65).

**FIG. 4.**
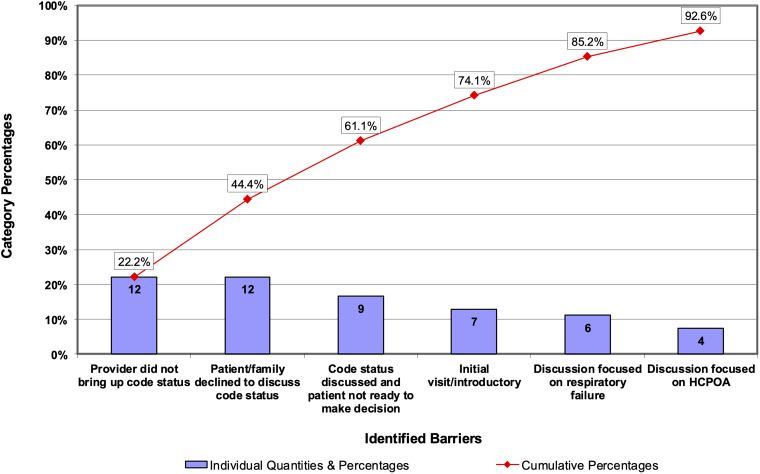
Pareto chart reviewing barriers to code status order documentation from September 2024 to March 2025.

A Pareto chart was constructed using data from September 2024 to March 2025 to better understand barriers to placing code status orders (Fig. 4). Analysis suggested that in many of these cases, code status was not discussed at all (22%), or if the conversation was initiated, patients declined to proceed with the discussion (22%). Other factors included patients not feeling ready to commit to a code status (7%), initial visits structured as an introduction to palliative care (11%), and discussions focused on chronic respiratory failure/tracheostomy decision-making (13%) or HCPOA completion (17%).

## Discussion

As patients with DMD enter early adulthood, they are not only learning the skills necessary to take ownership of their care but also facing new medical challenges and grappling with their mortality. Despite the progressive nature of DMD, there is limited research on the best approaches to ACP for this population. Integration of subspecialty palliative care within the multidisciplinary neuromuscular clinic was implemented as a way of supporting patients, families, and providers during a transitional period. The global aim of this QI project was to leverage the embedded palliative care provider to improve ACP and provide goal-concordant care for patients with DMD.

Through an iterative process, key components of disease-specific ACP and emergency planning were developed and integrated into a DMD ACP Checklist (Table 1). The checklist was a critical intervention as it served as an EHR-embedded tool to guide disease-specific conversations. Early discussions with patients explored value systems, communication preferences, and the people who support them in health care decision-making. During this process, many patients were able to identify a person or people to designate as HCPOA. Patients were then asked to consider potential future decisions that they or their surrogate may face, such as respiratory failure, heart failure, and code status. The team collaborated with subspecialists to share information that might guide decision-making during their visits and arranged joint visits when appropriate.

Because conversations were individualized and evolved differently for each patient, two concrete components were chosen to track as outcome measures: HCPOA completion and code status documentation. Rates of advance directive completion in young adults with neuromuscular disorders fall between one-quarter and one-third.^4^ Literature also suggests that ACP for adults with other chronic conditions of childhood such as congenital heart disease and cystic fibrosis ranges from 18% to 61%.^17–18^ Although DMD guidelines recommend that all patients age 18 and older have advance directives, a goal of 70% was selected to balance guideline recommendations with learnings from other chronic conditions of childhood and patient preference to choose not to complete ACP.^3^

Surprisingly, palliative care provider recognition of the value of both documenting a Full Code status and completing HCPOA paperwork required a culture change. Many young adults being cared for at a pediatric hospital have an illness of childhood, with their parents acting as legal decision makers until they turn 18. Assumptions are made that when patients reach adulthood, they will continue to choose their parents—who are by default the legal next of kin—to be their HCPOA, so completing paperwork had not been a priority. Additionally, at this institution, patients are treated automatically as Full Code if no code status is ordered in the chart. Thus, code status conversations are not routinely held in the inpatient or emergency department settings as is done in many adult institutions. Even if a patient explicitly chooses a Full Code status, this is not always ordered in the EHR. Education to reframe the value of completion of HCPOA and code status orders as part of ACP was a critical, although not highly reliable, part of obtaining palliative care team buy-in. Cases that validated the importance of ACP conversations included patients who selected only one parent as HCPOA or identified a different family member or friend instead. Other patients had hospital encounters where documentation noted a previously stated code status as helpful for guiding treatment. These real-life examples were critical in solidifying culture shift.

Another critical intervention was protocolization of the workflow for completing HCPOA paperwork in the clinic when patients expressed interest. An e-mail from the QI team served as a real-time reminder of the goals of the clinic visit and to provide state-specific HCPOA forms. Patients were noted to be more likely complete HCPOA paperwork if actively reviewed with them by a provider. This was a significant change in practice from simply sharing information about HCPOA paperwork or giving a copy to review at home. The team worked to ensure that every patient was at least given the state-specific paperwork at their first palliative care visit and that it was actively reviewed either at the initial visit or at a subsequent visit based on patient preference. This project helped to clearly define the palliative care provider as the team member who would discuss HCPOA paperwork and the neuromuscular clinic social worker as the team member who would witness completed forms. A remaining challenge includes the length of some state HCPOA forms that make them less feasible to complete during a clinic session.

Early on, it was identified that narrative notes did not consistently describe what parts of ACP conversations had been completed and what barriers to completion existed. Because providers within the neuromuscular clinic rotate, patients see different providers at each visit. This led to providers feeling like they were repeatedly reviewing the importance of ACP without making meaningful decisions. Feedback led to development of a DMD ACP Checklist that included disease-specific components of ACP and an indicator of patient readiness to proceed with ACP (Table 1). The checklist tool improved clarity of documentation and provided specific guidance on which components of ACP needed to be discussed and what paperwork needed to be completed. Importantly, it also acknowledged that different patients are ready to discuss components of ACP at different paces and provided guidance for providers to time visits appropriately to meet patient needs by spacing visits or coordinating co-visits with key subspecialists. While the team did not track individual components on the checklist aside from code status and HCPOA completion, documenting this information served as a useful tool for providers to understand what strategies were utilized for each individual patient. Overall, in addition to improving ACP completion, these interventions led to improved provider satisfaction, which made this intervention a key to success.

Several limitations were identified throughout the QI process. One limitation was that weekly e-mails from the QI team were a key component of successful implementation. To make the intervention more sustainable, verbal and written reminders were included during the daily team huddle, and access to state-specific HCPOA forms was improved through a shared online drive and in-office access to paper copies. The DMD ACP Checklist also now serves as a running EHR handoff within clinic notes (Table 1). Despite educational interventions targeting culture change on the palliative care team, other providers continued to have varying opinions on the importance of ACP. Specifically, current practice patterns and institutional standards, such as the lack of required code status documentation or the preference to use a parent as opposed to HCPOA as surrogate decision maker, may be more common due to the pediatric setting. However, current literature demonstrates that only one-third of adult patients complete advance directives, suggesting that pediatric hospital culture is not a unique barrier.^19^ Additionally, the study population was 91.1% non-Hispanic and white, compared with national DMD demographic data, where 61.7% of the population is non-Hispanic and white.^20^ With literature demonstrating racial and ethnic disparities in diagnosis and treatment of DMD, additional work is needed to explore potential barriers to accessing subspecialty referral centers.^20^ Finally, within the intervention group, only 49% of patients who self-reported HCPOA paperwork completion had documents scanned into the EHR. In this study, self-reported HCPOA paperwork was counted as a success; however, future directions should include improving workflows to ensure paperwork is obtained when completed outside of the hospital setting.

## Conclusions

This project demonstrated that by utilizing a QI framework, standardized embedded palliative care within a multidisciplinary clinic for young adults with DMD can improve completion of HCPOA and code status documentation as part of a broader framework of disease-specific ACP. Many patients were ready to engage in conversations surrounding HCPOA, while discussions surrounding code status were more difficult. Further work is required to understand how having these conversations and documenting preferences allows for goal-concordant care with illness progression.

## Authors’ Contributions

Both authors made substantial contributions to conceptualization, methodology, data collection, and data analysis, as well as drafting and editing the article.

## Abbreviations

ACP: Advance care planning
DMD: Duchenne muscular dystrophy
DNR: Do not resuscitate
EHR: Electronic health record
HCPOA: Health care power of attorney
NIPPV: Noninvasive positive pressure ventilation
QI: Quality improvement
SMART: Specific, measurable, achievable, relevant, time-based
